# Ophthalmological manifestations of COVID-19 and its transmissibility via ocular route

**DOI:** 10.1136/postgradmedj-2020-138069

**Published:** 2020-06-17

**Authors:** Aastha Gandhi, Mayuresh Naik, Anurag Narula

**Affiliations:** Ophthalmology, Vardhman Mahavir Medical College and Safdarjung Hospital, New Delhi, India; Ophthalmology, Hamdard Institute of Medical Science and Research, New Delhi, India; Ophthalmology, Vardhman Mahavir Medical College and Safdarjung Hospital, New Delhi, India

Coronavirus disease 2019 (COVID-19), declared a pandemic by the WHO on March 11, 2020, is caused by the highly transmissible Severe Acute Respiratory Syndrome Coronavirus 2 (SARS-CoV-2). The key to combating this disease besides prevention is rapid and early diagnoses of cases, which include identification of atypical presentations of this respiratory illness as well as possible routes of transmissibility. Recently, concerns regarding the eyes as both a portal of entry and a carrier of the virus have been raised, owing to the conjunctival signs and symptoms observed in a subset of patients with COVID-19 and the detection of SARS-CoV-2 in tears, thus presenting an additional risk potential for person-to-person transmission of this virus.

There have been several reports suggesting that SARS-CoV-2 can cause conjunctivitis, either as an early sign of infection or during hospitalisation for severe COVID-19 disease. In a meta-analysis by Loffredo *et al*^1^, they stated that the overall rate of conjunctivitis in confirmed patients with COVID-19 was 1.1%; it was 3% and 0.7% in severe and non-severe patients with COVID-19. They concluded that conjunctivitis is more frequent in severe COVID and may be a warning sign of poor outcomes. On the contrary, in a prospective conducted by Hong *et al*,^2^ they noticed that 15 (27%) of the 56 confirmed patients with COVID-19 had aggravated ocular symptoms, of which 6 (11%) had prodromal ocular symptoms before disease onset. They concluded that ocular symptoms are relatively common in COVID-19 and may appear just before the onset of respiratory symptoms. In a series of five cases, conjunctivitis as the sole presenting sign and symptom of COVID-19, with no other systemic manifestation of the illness, has also been reported.^3^ This has been supported by molecular evidence of the expression of ACE2 and TMPRSS2 in the conjunctiva, limbus and cornea,^4^ which are key factors required for cellular susceptibility to SARS-CoV-2 entry/infection proving that ocular surface cells are susceptible to coronavirus infection. In fact, in the conjunctiva, SARS-CoV-2 replication has been found to be much greater than SARS-CoV.^5^

Examination findings are usually indistinguishable from mild follicular conjunctivitis, including eyelid oedema, conjunctival congestion, watery discharge and follicular reaction of the palpebral conjunctiva.^6^ Although conjunctivitis is the most frequent ocular manifestation seen in patients with COVID-19, conjunctival hyperaemia, chemosis and epiphora have also been observed.^7^ Epiphora as the presenting symptom of COVID-19 has also been reported,^7^ and therefore ophthalmologists may be among the first to evaluate and hint a diagnosis of a patient with COVID-19.

Besides the ocular cells being a portal of entry, the isolation of SARS-CoV-2 in the tear samples in patients with confirmed COVID-19^7, 8, 9, 10^ has prompted concerns that a respiratory illness could be transmitted through ocular secretions as well as by fomite transmission when the infectious virus is introduced to the eyes via contaminated hands.^5^

However, the timing of appearance of SARS-CoV-2 in the conjunctival epithelium and tears is still uncertain and further studies are required to assess the same to assess the transmissibility by ocular route in early cases and most importantly in asymptomatic patients with COVID-19. This knowledge is not only essential among the front-line workers triaging what could be initial symptoms of COVID-19 but also could be a major source of transmission from patients with COVID-19 to healthcare workers and other people. Thus, protecting your mouth, nose (eg, using an N95 mask) and eyes (eg, goggles or breath shield) is recommended when caring for patients potentially infected with COVID-19 and as an extra precaution for the general public.^6^

## Supplementary Material

postgradmedj-97-329-DC1-inline-supplementary-material-1
