# Supplementary material for: Ophthalmological manifestations of COVID-19 and its transmissibility via ocular route
Source: Postgrad Med J. 2020 Jun 17;97(1147):329. doi: 10.1136/postgradmedj-2020-138069 (PMC10016920; doi:10.1136/postgradmedj-2020-138069)
Supplement: postgradmedj-97-329-DC1-inline-supplementary-material-1 [file postgradmedj-97-329-dc1-inline-supplementary-material-1.pdf]

<u>Ophthalmological manifestations of COVID-19 and its transmissibility via ocular route</u>

Dear Sir

Coronavirus disease 2019 (COVID-19), declared a pandemic by the WHO on March 11, 2020, is caused by the highly transmissible Severe Acute Respiratory Syndrome Coronavirus 2 (SARS-CoV-2). The key to combating this disease besides prevention is rapid and early diagnoses of cases, which includes identification of atypical presentations of this respiratory illness **as well as possible routes of transmissibility. Recently, concerns regarding the eyes, both as a portal of entry and carrier of the virus have been raised, owing to the conjunctival signs and symptoms observed in a subset of patients with COVID-19 and the detection of SARS-CoV-2 in tears, thus presenting an additional risk potential for person-to-person transmission of this virus.**

There have been several reports suggesting that SARS-CoV-2 can cause conjunctivitis, either as an early sign of infection or during hospitalization for severe COVID-19 disease. **In a meta-analysis by Loffredo et al [1], they stated that the overall rate of conjunctivitis in confirmed COVID-19 patients was 1.1%; it was 3% and 0.7% in severe and non-severe COVID- 19 patients. They concluded that conjunctivitis is more frequent in severe COVID and may be a warning sign of poor outcomes. On the contrary, in a prospective conducted by Hong et al [2], they noticed that fifteen (27%) of the 56 confirmed COVID-19 patients had aggravated ocular symptoms, of which six (11%) had prodromal ocular symptoms before disease onset. They concluded that ocular symptoms are relatively common in COVID-19 and may appear just before the onset of respiratory symptoms. In a series of 5 cases, conjunctivitis as the sole presenting sign and symptom of COVID-19, with no other systemic manifestation of the illness has also been reported. [3] This has been supported by molecular evidence of the expression of**

ACE2 and TMPRSS2 in the conjunctiva, limbus, and cornea [4], which are key factors required for cellular susceptibility to SARS-CoV-2 entry/infection proving that ocular surface cells are susceptible to coronavirus infection. Infact, in the conjunctiva, SARS-CoV-2 replication has been found to be much greater than SARS-CoV [5]

Examination findings are usually indistinguishable from mild follicular conjunctivitis, including eyelid edema, conjunctival congestion, watery discharge and follicular reaction of the palpebral conjunctiva. [6] Although conjunctivitis is the most frequent ocular manifestation seen in COVID patients, conjunctival hyperaemia, chemosis and epiphora have also been observed. [7]. Epiphora as the presenting symptom of COVID-19 has also been reported [7], so ophthalmologists may be amongst the first to evaluate and hint a diagnosis of a patient with COVID-19.

Besides the ocular cells being a portal of entry, the isolation of SARS-CoV-2 in the tear samples in patients with confirmed COVID-19 [7- 10], has prompted concerns that a respiratory illness could be transmitted through ocular secretions as well as by fomite transmission when infectious virus is introduced to the eyes via contaminated hands[5].

However the timing of appearance of SARS-CoV-2 in the conjunctival epithelium and tears is still uncertain and further studies are required to assess the same to assess the transmissibility by ocular route in early cases and most importantly, in asymptomatic COVID patients. This knowledge is not only essential amongst the front lines workers triaging what could be initial symptoms of COVID-19, but also could be a major source of transmission from COVID patients to healthcare workers and other people. Thus, protecting your mouth, nose (e.g., using an N95 mask) and eyes (e.g., goggles or breath shield) is recommended when caring for patients potentially infected with COVID-19 and as an extra precaution for the general public. [6]

REFERENCES

1. Loffredo, L., Pacella, F., Pacella, E., et al. Conjunctivitis and COVID- 19: a meta-analysis. *J Med Virol* 2020. doi:10.1002/jmv.25938

2. Hong, N., Yu, W., Xia, J et al. Evaluation of ocular symptoms and tropism of SARS-CoV- 2 in patients confirmed with COVID- 19. *Acta Ophthalmol* 2020. doi: 10.1111/aos.14445

3. Scalinci SZ, Trovato Battagliola E. Conjunctivitis can be the only presenting sign and symptom of COVID-19. *IDCases* 2020;20:e00774. doi:10.1016/j.idcr.2020.e00774

4. Headley C. COVID-19 found to be spread through eyes and is 100 times more infectious than SARS [Internet]. Ladders | Business News & Career Advice. 2020 [cited 28 May 2020]. Available from: https://www.theladders.com/career-advice/covid-19-found-to-be-spread-through-eyes-and-is-100-times-more-infectious-than-sars

5. Hui K P Y, Cheung M-C, Perera R A P M, et al. Tropism, replication competence, and innate immune responses of the coronavirus SARS-CoV-2 in human respiratory tract and conjunctiva: an analysis in ex-vivo and in-vitro cultures. *Lancet Respir Med* 2020. doi: 10.1016/S2213-2600(20)30193-4

6. Hu K, Patel J, Patel BC. Ophthalmic Manifestations Of Coronavirus (COVID-19) [Updated 2020 Apr 13]. In: StatPearls [Internet]. Treasure Island (FL): StatPearls Publishing; 2020 Jan-. Available from: https://www.ncbi.nlm.nih.gov/books/NBK556093/

7. Wu P, Duan F, Luo C, et al. Characteristics of Ocular Findings of Patients With Coronavirus Disease 2019 (COVID-19) in Hubei Province, China. *JAMA Ophthalmol* 2020. doi:10.1001/jamaophthalmol.2020.1291

8. Xia J., Tong J., Liu M, et al. Evaluation of coronavirus in tears and conjunctival secretions of patients with SARS-CoV-2 infection. *J Med Virol* 2020 doi: 10.1002/jmv.25725. Feb 26 [Online ahead of print].

9. Zhang X1, Chen X1, Chen L1, et al. The evidence of SARS-CoV-2 infection on ocular surface. *Ocul Surf* 2020 doi: 10.1016/j.jtos.2020.03.010. [Epub ahead of print]

10. Zhou Y, Duan C, Zeng Y, et al. Ocular Findings and Proportion with Conjunctival SARS-COV-2 in COVID-19 Patients [published online ahead of print, 2020 Apr 21]. *Ophthalmology* 2020;S0161-6420(20)30405-X. doi:10.1016/j.ophtha.2020.04.028
